# Hypoxia signaling pathways in cancer metabolism: the importance of co-selecting interconnected physiological pathways

**DOI:** 10.1186/2049-3002-2-3

**Published:** 2014-02-04

**Authors:** Norma Masson, Peter J Ratcliffe

**Affiliations:** 1 The Hypoxia Biology Laboratory, The Henry Wellcome Building for Molecular Physiology, The University of Oxford, Roosevelt Drive, Oxford OX3 7BN, UK

**Keywords:** Hypoxia, HIF, Metabolism, Cancer

## Abstract

Both tumor hypoxia and dysregulated metabolism are classical features of cancer. Recent analyses have revealed complex interconnections between oncogenic activation, hypoxia signaling systems and metabolic pathways that are dysregulated in cancer. These studies have demonstrated that rather than responding simply to error signals arising from energy depletion or tumor hypoxia, metabolic and hypoxia signaling pathways are also directly connected to oncogenic signaling mechanisms at many points. This review will summarize current understanding of the role of hypoxia inducible factor (HIF) in these networks. It will also discuss the role of these interconnected pathways in generating the cancer phenotype; in particular, the implications of switching massive pathways that are physiologically 'hard-wired’ to oncogenic mechanisms driving cancer.

## Review

Altered energy metabolism is a classical feature of cancer that underpins the diagnostic use of labeled fluorodeoxyglucose in positron emission tomography (FDG-PET) and is the focus of major efforts to define new therapeutic approaches to the disease. In normal cells, glucose is converted through the Embden-Meyerhof glycolytic pathway to pyruvate, which is then predominantly fed into the mitochondrion for ATP production. Cancer cells exhibit an enhanced capacity to 'ferment’ glucose to pyruvate and then lactate, even in the presence of sufficient oxygen to support mitochondrial metabolism. This effect was first described almost 90 years ago by Warburg, who became convinced that the primary defect was in mitochondrial function, famously stating (of cancer) 'the respiration is always disturbed inasmuch as it is incapable of causing the disappearance of the fermentation’ [1]. Later reviewers of the controversy have puzzled over why Warburg apparently held this view so firmly, particularly as it was not clearly supported by his own data on oxygen uptake [2]. More in keeping with current thinking, Warburg also stated of glycolysis that it *'*furnishes, to our mind, the driving forces of growth’ [3]. However, it became clear, not only that mitochondrial capacity in many cancers is broadly comparable with normal tissue, but that up-regulation of glycolysis is not universal in cancer [2]. Studies of experimental tumors revealed that aerobic glycolysis was not greatly elevated in slow growing tumors, despite their potential to metastasize and kill the host, but was strongly associated with poorly differentiated rapid growing variants. This change was revealed to be strikingly associated with a shift in gene expression to particular enzyme isoforms that are characteristic of fetal tissues [4,5].

Despite these observations, in the latter part of the 20th century, the focus on metabolism in cancer was overshadowed by genetic insights into oncogenesis that implicated molecules directly involved in the regulation of the cell cycle, DNA repair, growth, apoptosis and related processes. It also became clear that the metabolic dysregulation described by Warburg was not entirely specific for cancer, being observed, for instance, in dividing cells in immune activation [6,7]. Although this led to the refocusing of cancer research on oncogene and tumor suppressor pathways that had been identified genetically, there has been a major resurgence of interest in understanding metabolic pathways in cancer, including the mechanisms responsible, the means by which these changes support tumor development and whether this offers a route to treatment.

Alongside metabolic alterations, tumor hypoxia and activation of hypoxia signaling pathways have consistently been identified as features that are strongly associated with aggressive malignancy. Hypoxia inducible factor (HIF) has been defined as the key transcription factor mediating responses to hypoxia, and HIF target genes overlap strongly with those implicated in dysregulated tumor metabolism. This review will focus on the interfaces between the HIF system and metabolic alterations in cancer and on the implications of activating complex, interconnected, hypoxia and metabolic signaling pathways for the cancer phenotype.

### The HIF hydroxylase system

Hypoxia inducible factor-1 (HIF-1) was first identified as a transcriptional regulator that is bound to the hypoxia-response element of the erythropoietin gene [8]. It was initially believed that the underlying oxygen sensing apparatus was restricted to the regulation of erythropoietin. However, early studies of the hypoxia-response element unexpectedly indicated that the response pathway operated much more widely [9]. It subsequently became clear that HIF-1 has a wide range of other transcriptional targets, the first to be identified being genes encoding a range of glycolytic enzymes [10,11]. Interestingly, these studies revealed that regulation by HIF was specific to particular enzyme isoforms and that the isoform-specific pattern of regulation by HIF was strikingly similar to that which had been identified earlier in analyses of the Warburg effect in cancer cells, immediately raising a question as to the role of HIF in metabolic alterations in cancer [12].

**Figure 1:**
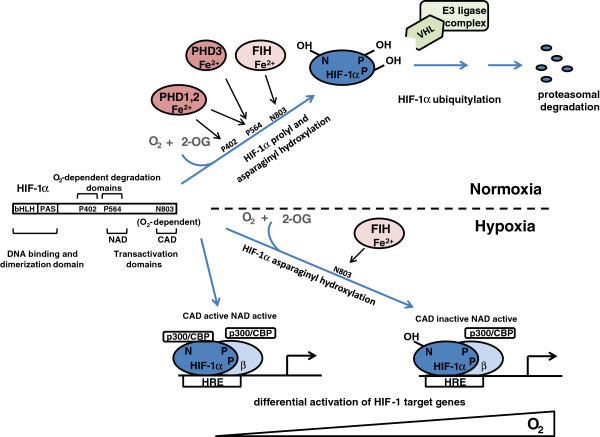
**Regulation of HIF-1 by oxygen dependent prolyl and asparaginyl hydroxylation of HIF-1α.** Hypoxia inducible factor (HIF)-1α, a basic-Helix-Loop-Helix Per-AHR/ARNT/Sim (bHLH-PAS) domain containing protein, contains three residues that are targets for regulatory hydroxylation. P402 and P564 are targeted by the prolyl hydroxylase domain (PHD) enzymes (note that PHD3 can only hydroxylate P564) and N803 by factor inhibiting HIF (FIH). P402 is located in the N-terminal, and P564 in the C-terminal, O_2_-dependent degradation domain. Prolyl hydroxylated HIF-1α is recognized by the von Hippel-Lindau tumor suppressor (pVHL) E3 ligase complex, leading to degradation in normoxia. Interestingly, prolyl and asparaginyl hydroxylation are differentially sensitive to hypoxia. Inhibition of prolyl hydroxylation alone (lower right) is sufficient to allow HIF-1α to escape from pVHL E3-dependent proteolytic destruction and form an active transcriptional complex with HIF-β through activity of the N-terminal activation domain (NAD). In more severe hypoxia, HIF-1α asparaginyl hydroxylation is also inhibited (lower left) allowing recruitment of p300/CBP co-activators to its C-terminal transactivation domain (CAD), and enhancing the transcription of a specific set of HIF-1 target genes. (HRE, hypoxia-response element).

The HIF system itself has been reviewed extensively elsewhere (for more detailed reviews see [13,14]). In outline, HIF is a heterodimer of α and β subunits, both of which are bHLH-PAS domain (basic-Helix-Loop-Helix Per-AHR/ARNT/Sim) proteins. Three HIF-α isoforms exist (1α, 2α and 3α), of which HIF-1α and HIF-2α are the best studied, and form transcriptionally active heterodimers with HIF-1β. Hypoxia inducible behavior is conferred by the HIF-α subunits, the protein abundance and transcriptional activity of which are regulated by oxygen-dependent prolyl and asparaginyl hydroxylation respectively (Figure 1).

The most essential of these processes is HIF prolyl hydroxylation, which exerts the most important control over HIF activity in human cells, and is conserved in all animal species. In higher animals, HIF prolyl hydroxylation occurs at two sites (P402 and P564 in human HIF-1α). Hydroxylation at these sites promotes association of HIF-α polypeptides with the β-domain of von Hippel-Lindau tumor suppressor (pVHL) E3 ligase, leading to the destruction of HIF-α by the ubiquitin-proteasome pathway. Though the two sites of prolyl hydroxylation can operate independently, in native HIF-1α and HIF-2α molecules these sites are hydroxylated sequentially and cooperate to enhance the efficiency of pVHL-mediated proteolysis. In a second regulatory pathway, asparaginyl hydroxylation (N803 in human HIF-1α) reduces the activity of the C-terminal transactivation domain (CAD), at least in part by preventing recruitment of the p300/CBP co-activators (reviewed in [13]).

**Figure 2:**
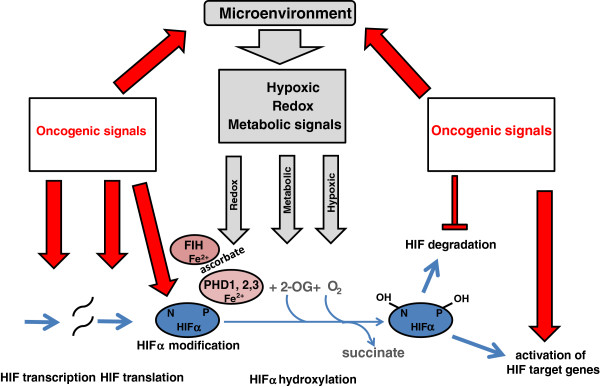
**Oncogenic signals act through multiple parallel pathways to activate HIF and its target genes.** In addition to regulation by hypoxia, hydroxylation of hypoxia inducible factor (HIF)-α may be influenced by metabolic and redox signals. In cancer, all of these micro-environmental stresses can inhibit hydroxylation leading to accumulation of HIF. Oncogenic signals also impinge on the HIF pathway at many other points, including transcription, translation, post-translational modification and pVHL-mediated degradation of HIF-α polypeptides. In addition, oncogenic signals activate many HIF target genes directly.

These hydroxylations are all catalyzed by members of the Fe(II) and 2-oxoglutarate (2-OG) dependent dioxygenase superfamily (for reviews, see [15,16]). As dioxygenases, the enzymes split molecular oxygen and incorporate both atoms directly into their reaction products. The absolute requirement for molecular oxygen as a substrate confers sensitivity to hypoxia, though this may be modulated by other factors. Oxidation of the prime substrate (HIF-α) is coupled to the oxidative decarboxylation of 2-OG to succinate. Failure of the coupling process can leave the enzyme in an inactive oxidized state and regeneration of the Fe(II) catalytic center is then required for activity. This process is proposed to be the basis of the dependency on the reducing agent ascorbate, which is required to regenerate the active Fe(II) enzyme. Co-ordination of the catalytic iron is relatively labile, hence enzymes are iron-dependent and catalysis is readily inhibited by iron chelators. In human cells, HIF prolyl hydroxylation is catalyzed by three closely related enzymes, PHD (prolyl hydroxylase domain) 1, 2 and 3 (otherwise known as EGLN 2, 1 and 3), while HIF asparaginyl hydroxylation is catalyzed by a single enzyme, FIH (factor inhibiting HIF). The multiple co-substrate and co-factor requirements of these enzymes (2-OG, Fe(II) and ascorbate, in addition to molecular oxygen) potentially allow regulation of HIF activity by redox, metabolic and hypoxic stimuli. In cancer, all these signals may contribute to up-regulation of HIF pathways, by impairing the activity of one or more of the HIF hydroxylases (Figure 2).

In addition, other interactions, which are not directly connected with the oxygen sensing process, but which modulate the HIF pathway through effects on the transcription, translation, post-translational modification and protein interactions of one or more HIF subunits, are also the targets of oncogenic processes that up-regulate HIF in cancer (Figure 2).

### Up-regulation of hypoxia signaling pathways in cancer

#### Tumor hypoxia

Regions of profound hypoxia and necrosis are common in solid tumors and their presence correlates with an aggressive clinical course. Classical studies relating necrotic regions of tumor to blood vessel disposition suggested that tumor necrosis was, at least in part, driven by hypoxia [17]. More recently, this has been supported by the demonstration of striking up-regulation of HIF target genes in regions immediately adjacent to the necrotic areas [18]. This pattern of gene expression is absent in experimental tumors that are genetically defective for HIF, clearly indicating that it is driven by this pathway [19].

Given that HIF target genes include many of those underpinning dysregulated tumor metabolism [20] and that both tumor hypoxia [21] and the extent of dysregulated metabolism [2,5] show clear correlations with aggressive cancer phenotypes, it is tempting to conclude that activation of HIF pathways by tumor hypoxia itself is the major cause of dysregulated tumor metabolism. However, a number of observations reveal that the interconnections are more complex. First, within solid tumors, regions of hypoxia (assessed either by *in vivo* imaging or by use of histochemical markers) show less co-incidence with regions of HIF up-regulation than might be expected [22-24]. Second, hematological malignancies that do not involve solid tissue masses also manifest up-regulation of HIF [25,26]. Taken together these studies indicate that although micro-environmental hypoxia clearly contributes to the activation of HIF in cancer, other factors must also be important.

#### Metabolic regulation of HIF pathways

Multiple metabolic pathways impact on the regulation of HIF, raising the possibility that in addition to HIF activation driving dysregulated metabolism in cancer, dysregulated metabolism promotes the activation of HIF. One possibility is that altered 2-OG availability modulates HIF hydroxylation. In addition to its role in the Krebs cycle, 2-OG also serves as a co-substrate/product in reductive amidation/oxidative deamidation by glutamate dehydrogenase and is the major amino group acceptor for transaminases. Thus, 2-OG would be well placed to act as a metabolic sensor regulating HIF hydroxylase activity. In keeping with this, reduced intracellular 2-OG in cultured cells depleted of amino acids has recently been reported to reduce PHD activity [27]. Unexpectedly, in this case, the regulation of PHD activity had no effect on HIF-α protein levels, but was reported to effect mTORC1 activation by amino acids. The PHD enzymes were suggested to play a role as metabolic sensors linking amino acid availability with the mTORC1 pathway, although whether limiting 2-OG availability modulates HIF hydroxylation in this setting remains unclear.

Other Krebs cycle intermediates and endogenous organic acid metabolites may also alter HIF hydroxylase activity by competing with 2-OG at the catalytic site (fumarate and succinate) or by product inhibition (succinate) [28,29]. In addition, in different assays, citrate, isocitrate, malate and oxaloacetate have been reported to bind or inhibit recombinant HIF hydroxylases. Different HIF hydroxylases are differentially sensitive to these inhibitors. For instance, fumarate is a more potent inhibitor of the PHDs than FIH, whereas citrate is a more potent inhibitor of FIH than the PHDs [28,30]. Both fumarate and succinate reach very high levels in hereditary cancers associated with the inactivation of fumarate hydratase (FH, hereditary leiomyomatosis and papillary renal cell carcinoma) and succinate dehydrogenase (SDH, hereditary paraganglioma) respectively [31-33]. In these settings, fumarate and succinate clearly induce HIF, at least in part by inhibiting the PHD enzymes [30]. However, whether and under what circumstances the Krebs cycle and other metabolic intermediates reach the levels required to inhibit the HIF hydroxylases in common cancers is less clear.

Interestingly, a number of studies has demonstrated that provision of glucose in cell culture medium and/or on-going glucose metabolism is necessary for the induction of HIF-1α by hypoxia [34]. Different investigators have provided evidence for a range of mechanisms. Provision of lactate or pyruvate has been shown to stabilize HIF-1α in glucose depleted cell cultures and it has been suggested that this effect is mediated by inhibition of the PHDs by pyruvate produced from lactate by lactate dehydrogenase [35]. Lactate often accumulates to high levels (10 mM) in tumors and has also been proposed to activate HIF and vascular endothelial growth factor (VEGF)-mediated angiogenesis in cancer [36]. Oddly, neither pyruvate nor lactate was found to compete with 2-OG or inhibit purified recombinant PHDs under standard (ascorbate containing) reaction conditions [28,29]. Some insight into this paradox may be provided by recent work suggesting a different mode of inhibition, whereby pyruvate and oxaloacetate inactivate the PHDs by oxidation, which is reversed by ascorbate [37]. Thus, metabolic intermediates have the potential to inhibit the HIF hydroxylases by at least two mechanisms; competition with 2-OG and oxidation.

Recently, interest has also focused on another 'oncometabolite’, 2-hydroxyglutarate (2-HG), which has the potential to competitively inhibit 2-OG dioxygenases. Specific mutations in the genes encoding isocitrate dehydrogenases (IDH) 1 and 2 have been observed at a high rate in low and medium grade gliomas, secondary glioblastoma, acute myeloid leukemia, and at a lower rate in other malignancies, including myelodysplastic syndromes, T-cell lymphoma, chondrosarcoma and cholangiocarcinoma [38,39]. In affected cells, 2-HG is formed as a result of reduction of 2-OG by the abnormal enzyme and accumulates to very high levels [40]. However, 2-HG, particularly the 'R’ enantiomer that is formed by the mutant IDH enzymes, is a poor inhibitor of the PHD enzymes [41]. It is therefore unlikely to contribute to any up-regulation of HIF that is observed in these settings and has even been reported to activate PHD2, resulting in a reduction in HIF [42].

In addition to effects of metabolites on the 'oxygen sensing’ hydroxylation reaction, multiple interactions of metabolic and HIF signaling pathways have been defined at other levels. For instance, HIF-α levels are subject to complex translational controls operating through nutrient and cellular energy-sensing mTOR complexes, with HIF-1α being regulated through mTORC1 and 2, and HIF-2α principally by mTORC2 [43]. In normal cells, several mechanisms exist whereby hypoxia can reduce translation either through mTOR pathways or via regulation of eIF2α or eEF2. These pathways are themselves independent of HIF and involve activation of AMPK or PERK in response to metabolic changes arising from hypoxia [44]. However, translational control can also be HIF-dependent. For instance, HIF activates transcription of REDD1 [45], which activates the tuberous sclerosis TSC1/2 tumor suppressor complex [46], an upstream inhibitor of mTORC1. Cancer cells can evade this down-regulation of HIF-α translation at least in part through oncogenic dysregulation of mTOR complexes.

Yet another interface with metabolism is mediated by reversible acetylation at specific sites in HIF-α polypeptides. HIF-1α can be acetylated at multiple lysine residues by acetyltransferases, such as p300/CBP associated factor (PCAF), and acetylation can be reversed by several classes of enzyme, including classical histone deacetylases (HDACs) and sirtuins [47-49]. Given their sensitivity to another key parameter of energy status, the cellular NAD+/NADH ratio, the interface between sirtuins and HIF has attracted widespread interest. The mammalian sirtuin family (SIRT1-SIRT7) of lysine deacetylases couple deacetylation with NAD + hydrolysis. SIRT1, 3 and 6 have all been implicated in the regulation of HIF activity [48,50-53], although there is disagreement as to the exact nature of the interconnections. In one study, deacetylation of HIF-1α at K674 by SIRT1 was reported to block p300 recruitment by HIF-1α, with the inactivation of SIRT1 in hypoxia (by decreased NAD + levels) releasing this negative control [48]. Another study has reported SIRT1 activity to be required for full activity of HIF-1α in hypoxia [53], while a third reports a specific functional interaction between SIRT1 and HIF-2α leading to up-regulation of HIF-2α transactivation in hypoxia [52]. SIRT1 itself is also reported to be a HIF target gene in some [54], but not all, settings [53].

#### Iron, ascorbate and oxidant stresses

The binding of Fe(II) at the catalytic center of the HIF hydroxylases, like that of other 2-OG dioxygenases, is relatively labile. The enzymes also require ascorbate for maintenance of an active Fe(II) catalytic center. These properties render them susceptible to modulation by redox signals and iron availability, raising questions as to whether abnormalities in redox status and/or iron availability provide another link between abnormal metabolism in rapidly dividing cancer cells and activation of HIF.

In tissue culture, supplementation with either iron or ascorbate promotes HIF hydroxylase activity and suppresses basal HIF levels in oxygenated cells [55]. Cancer patients are often poorly nourished and systemic iron deficiency is common [56]. Furthermore, rapid growth and/or poor blood supply may exacerbate cellular iron and ascorbate deficiencies within the tumor. Somewhat surprisingly, the possibility that iron and ascorbate deficiency may be important contributors to HIF activation in clinical cancer has not been intensively investigated. In scorbutic rodents, ascorbate supplementation did not affect physiological measures of HIF activation, such as the production of erythropoietin, suggesting that tissue culture studies may not be representative of effects in the intact organism [57]. Nevertheless, low cellular ascorbate has recently been associated with increased HIF and aggressive phenotype in clinical endometrial cancer [58].

The ability of both iron deficiency and redox stresses to up-regulate HIF in tumors is strongly supported by experimental studies. For instance, the suppression of iron uptake by shRNA-mediated knockdown of transferrin receptor-1 has been shown to activate HIF and enhance angiogenesis in a breast cancer cell line xenograft model [59]. In tumors derived from Ki-Ras transformed fibroblasts, activation of the antioxidant response by junD has been reported to enhance PHD activity, reduce HIF and impair angiogenesis [60]. In another xenograft model, both ascorbate and the anti-oxidant N-acetylcysteine were found to suppress HIF activation and growth of human lymphoma cells through increased hydroxylation [61]. Kinetic studies on purified recombinant enzyme have indicated that reducing agents other than ascorbate can only partially substitute for ascorbate in activating the HIF hydroxylases [62], and it is possible that in cells, these agents are acting indirectly on processes that affect cellular Fe(II) or ascorbate levels.

It has also been proposed that the increased production of mitochondrial reactive oxygen species in hypoxia contributes to HIF activation by impairing the activity of HIF hydroxylases (reviewed in [63]). However, whether reduced activation of HIF following the application of mitochondrial inhibitors arises from a reduction in reactive oxygen species, or an increase in intracellular oxygen levels (as a result of reduced mitochondrial oxygen consumption) is controversial (reviewed in [64]). Interestingly, HIF asparaginyl hydroxylation is much more sensitive to inhibition by hydrogen peroxide than HIF prolyl hydroxylation [65], whereas the reverse is true for inhibition by hypoxia [66]. This suggests that hypoxia and reactive oxygen species affect HIF signaling by distinct mechanisms. Since HIF asparaginyl hydroxylation persists under all but the most severe levels of hypoxia [66] and specifically modulates the expression of some but not all HIF target genes [67], these findings also suggest that interplay between hypoxia and redox signals in tumors not only activates HIF, but shapes the nature of the HIF transcriptional response.

In addition to modulation of HIF hydroxylase activity, redox signals and oxidant stress (such as metabolic dysregulation) impinge on the HIF pathway at many other levels. Effects are observed on both transcription and translation of individual HIF-α isoforms. For instance, the HIF-1α promoter contains a well characterized nuclear factor kappa B (NF-kB) binding site that conveys up-regulation by oxidant stresses [68], while HIF-1β transcription can also be activated directly by NF-kB [69]. Studies of the effects of nicotinamide adenine dinucleotide phosphate (NADPH) oxidases in pVHL-defective renal cancer cell lines (in which the pVHL-dependent proteolysis is disrupted) suggest several other levels of control. For instance, NADPH oxidase, specifically Nox4, was found to elevate HIF-2α mRNA levels [70], whereas NADPH oxidase-dependent generation of reactive oxygen species has been proposed to enhance translation of HIF-2α [71]. Taken together, these findings reveal multiple interactions with redox signals that have the potential to affect both quantitative and qualitative aspects of HIF pathway activation in cancer.

#### Oncogenic and tumor suppressor pathways

In addition to activation by multiple micro-environmental stimuli, the HIF system is activated by diverse tumor suppressor and oncogene pathways. The most striking of these is mutation of the von Hippel-Lindau tumor (*VHL*) suppressor (reviewed in [72]). As outlined above, pVHL is part of the ubiquitin E3 ligase complex that targets HIF-α subunits to the ubiquitin-proteasome pathway. Biallelic inactivation of *VHL* thus blocks oxygen-dependent proteolysis of HIF-α and leads to constitutive activation of the HIF pathway. Interestingly, however, more detailed analysis of HIF in pVHL-associated cancer has revealed the importance of quantitative effects on HIF activation. In particular, there is a clear correlation between the quantitative effects of specific mutations on HIF dysregulation and the prevalence of different types of neoplasia in families affected by VHL disease [73,74]. Severe dysregulation of HIF is associated with a predisposition to renal cancer, but appears to be incompatible with pVHL-associated phaeochromocytoma, which is associated with partially inactivating mutations that lead to more modest levels of HIF pathway activation.

HIF is also activated by a range of growth factors acting through PI3K/PTEN/AKT or RAS/RAF/MAPK signaling cascades (reviewed in [75]). Activation of these pathways by somatic mutation and gene amplification is common in many types of cancer and dysregulation of the PI3K/PTEN/AKT pathway leads to up-regulation of HIF through increased synthesis of HIF-α subunits [76]. The AKT serine/threonine kinase has multiple downstream targets, and likely increases HIF-α translation by both mTOR-dependent and mTOR-independent mechanisms [77]. It is also possible that AKT may increase HIF-α levels through other mechanisms. For example, another substrate of AKT, GSK3b, has been implicated in regulating HIF-1α protein degradation through a pVHL independent mechanism [78].

The RAS/RAF/MAPK pathway has been reported to impact on HIF activity primarily through the regulation of transactivation. Phosphorylation of either HIF-1α or the co-activator p300 by different kinases (either p42/p44 MAPK or p38) activates HIF, both by promoting the formation of HIF/p300 complexes and by enhancing p300 transactivation [79].

Diverse interactions between HIF and p53 tumor suppressor pathways have been reported (reviewed in [80,81]). Though not all reports are in agreement, the induction of p53 has generally been shown to suppress HIF activity. Both direct physical interactions between p53 and HIF-1α [82] and indirect functional interactions have been described, including competition between p53 and HIF-α for the p300 co-activator [83] and p53-dependent promotion of HIF-α degradation by the mouse double minute 2 homolog (MDM2) ubiquitin-ligase [84].

#### Genetic mutation

In contrast with the prevalence of mutations in classical tumor suppressor and oncogenic pathways that are linked to the HIF system, direct mutational activation of HIF (for example, by deletion or mutation of key residues in the degradation domain) is not common in cancer. Rather, in pVHL-defective renal cancer, a small but significant excess of inactivating mutations has been observed in HIF-1α and reduction in HIF-1α gene dosage through deletion of a region of chromosome 14q is common [85-87]. Inactivating mutations in the HIF hydroxylases are also uncommon in cancer.

Taken together these findings reveal a plethora of means and mechanisms by which both micro-environmental and genetic alterations lead to the up-regulation of HIF in cancer. However, they also demonstrate a remarkable contrast between the low prevalence of cancer-associated activating mutations in HIF itself and the high prevalence of mutations in multiple oncogenes and tumor suppressor genes whose products impinge on the activity of the pathway.

### Regulation of metabolism by HIF

#### Glycolysis

Following the identification of genes encoding glycolytic enzymes as the first non-erythropoietin targets of the HIF system [10,11] the role of HIF in the glycolytic pathway has been extensively studied, at least at the level of gene expression. These studies have established an action of HIF at almost every step in glycolysis. Both the specific glucose transporters necessary for the initial glucose internalization, and monocarboxylic acid transporters that promote lactate efflux, have been identified as HIF-1 target genes [88,89]. Thus, HIF impacts on both the glycolytic pathway itself and on the ancillary processes that support it. HIF-dependent transcription is strikingly isoform or isoenzyme specific. For instance, hypoxia up-regulates lactate dehydrogenase A and monocarboxylate transporter 4 (which act to promote conversion of pyruvate to lactate and lactate efflux from the cell), but down-regulates monocarboxylate transporter 1 and lactate dehydrogenase B (which act to promote lactate uptake and conversion to pyruvate in the reverse direction) [89].

The glycolytic isoenzymes that are up-regulated by HIF are remarkably similar to those that are over-expressed in cancer cells. In general, they have kinetic properties that enhance flux through particular components of the glycolytic pathway, or are less sensitive to inhibitory regulators (reviewed in [90]). This is illustrated by 6-phosphofructo-1-kinase (PFK-1), which catalyzes a key regulatory step in glycolysis, conversion of fructose-6-P to fructose-1,6-bisphosphate. PFK-1 exists as three isoforms (PFK-L, -P and -M), which differ in their sensitivity to feedback inhibition by ATP and the Krebs cycle intermediate citrate. The PFK-L isoform, which is the principal isoform up-regulated by HIF, is the least sensitive to these inhibitors. However, although PFK-L is up-regulated by HIF-1, its activity remains allosterically controlled by fructose-2,6-bisphosphate, the product of a dual kinase/phosphatase family of enzymes (6-phosphofructo-2-kinase/fructose-2,6-bisphosphatase 1-4, PFKFB1-4). Binding of fructose-2,6-bisphosphate promotes PFK tetramer formation and increases catalytic activity. HIF-1 also induces expression of PFKFB enzymes; in particular, the PFKFB-3/4 isoforms that have a high ratio of kinase to phosphatase activity and hence increase fructose-2,6-bisphosphate levels [91]. Yet further complexity is added by post-translational regulation of the ability of PFK-1 to bind fructose-2,6-bisphosphate. Glycosylation (O-linked β-N-acetylglucosamine) at serine 529 prevents binding of fructose-2,6-bisphosphate, inhibits activity of PFK-1, and is proposed to redirect flux to the biosynthetic pentose phosphate pathway [92]. Although the role of HIF was not studied, glycosylation at serine 529 was reported to be strongly induced by hypoxia, apparently opposing the effect of HIF in inducing fructose-2,6-bisphosphate levels.

Another important regulatory enzyme is pyruvate kinase. Pyruvate kinase catalyzes the terminal step in glycolysis, and is therefore well placed to alter the metabolic fate of glucose: either to maximize ATP generation; or to slow glycolysis, resulting in a build-up of glycolytic intermediates that supply biosynthetic pathways. HIF-1 induces transcription of the *PKM* gene [93]. However cancer cells replace the normal form of pyruvate kinase (PKM1) with an alternatively spliced embryonic form (PKM2) which is less active. The conversion to PKM2 alters the residues within the major inter-subunit contact domain [94] and makes PKM2 activity more readily down-regulated by reversible subunit dissociation from active tetramer to inactive dimer. The switch to PKM2 also facilitates catalysis of phosphoenolpyruvate (PEP)-dependent histidine phosphorylation of the upstream enzyme phosphoglycerate mutase-1 (PGAM-1), which increases PGAM-1 activity and is again proposed to redirect glycolytic flux away from ATP synthesis and into the production of biosynthetic intermediates [95]. Interestingly, the role of PKM2 in supporting cancer growth appears to be highly context specific. Indeed, knockdown of PKM2 yielded contradictory results in xenograft tumor models [96,97] and a recent study of tumorigenesis in mice has revealed that while PKM2 isoform specific deletion enhances tumor growth, it also appears to select against outgrowth of tissue culture cell lines from the tumors [98]. Whether HIF is involved in the alternative splicing process that switches PKM1 to PKM2, as opposed to up-regulation of the primary transcript is not yet known. Interestingly, however, a quite different, but specific, interaction of PKM2 with the HIF system has been proposed in a recent report describing a non-glycolytic role of PKM2 in the transcriptional co-activation of HIF-1α, potentially driving a positive reinforcing circuit [93].

Taken together with the proposed action of glycolytic intermediates in the regulation of HIF, these findings indicate the existence of an extremely complex interplay between the HIF pathway and glycolysis. However, relatively few studies have actually measured HIF-dependent changes in metabolic fluxes, as opposed to levels of enzymes and regulators. Studies of transformed mouse embryonic fibroblasts have demonstrated that in hypoxic culture, inactivation of HIF-1α is associated with reduced lactate production and impaired maintenance of ATP levels [99]. Thus, down-regulation of HIF by oxygen appears, at least in part, to contribute to the 'Pasteur effect’ in mammalian cells (down-regulation of fermentation in the presence of oxygen). Interestingly, however, these studies did not reveal an effect of HIF-inactivation on lactate production in normoxic cells. Similarly, recent metabolic profiling of Hct116 colon cancer cells revealed that while siRNA mediated knock-down of HIF-1β reduced hypoxic up-regulation of lactate production, it did not reduce glucose uptake in either normoxic or hypoxic cells [100]. Furthermore, studies of HIF-1β deficient hepatoma cells have revealed that glycolytic flux as assessed by FDG-PET analysis of glucose uptake and by measurement of lactate output was maintained, despite down-regulation of multiple glycolytic genes in the absence of HIF. Further analysis indicated that the enhanced glycolytic flux was associated with allosteric activation of PFK-1 by an increase in the AMP/ATP ratio in the HIF-1β deficient cells [101].

Thus, although the activation of HIF in cancer cells and the action of HIF on specific glycolytic enzymes, regulators and transporters would all suggest that HIF makes an important contribution to the Warburg effect, intervention studies have revealed that up-regulated glycolysis continues at least in some settings in the absence of HIF. Studies of the action of tumor suppressors and oncogenes have revealed multiple direct interfaces with the regulation of glycolysis and parallel pathways of oncogenic glycolytic activation, presumably accounting for these observations. Nevertheless, the isoenzyme specific targeting by HIF of essentially every point in the glycolytic pathway strongly suggests that HIF contributes, albeit through parallel pathways, to the up-regulation of glycolysis in cancer.

#### Pentose phosphate pathway

In most cells, in addition to entering glycolysis, glucose-6-phosphate can also be metabolized by the pentose phosphate pathway which, through a series of cytoplasmic reactions, generates NADPH (used in reductive synthesis of fatty acids and sterols), ribose-5- phosphate (used in synthesis of nucleotides and nucleic acids) and other biosynthetic intermediates. Ribose-5-phosphate has been identified in metabolic profiling of cancer cells as one of the most strongly up-regulated metabolites in hypoxia [100]. Whether this reflects changes that limit flux through glycolysis, such as glycosylation of PFK-1 (see above) or changes in the pentose phosphate pathway itself is unclear. Interestingly, however, glucose-6-phosphate dehydrogenase, the enzyme which catalyzes the entry step to the pentose-phosphate pathway, is up-regulated by hypoxia in at least some cancer cells, though whether this is as a response to HIF has not been resolved [102].

#### Glycogen metabolism

Studies of glycogen metabolism also support a key role for HIF in biosynthetic metabolic pathways that are dysregulated in cancer. Increased storage of glycogen is a common feature of cancer cells, which, along with lipid deposition, contributes to the clear cell phenotype in some tumors. As with the glycolysis, enzymes catalyzing multiple steps in glycogen biosynthesis have been identified as HIF target genes, including phosphoglucomutase 1 (the first enzyme in the pathway), UDP-glucose pyrophosphorylase 2 (which forms UDP-glucose, the direct precursor of glycogen), glycogen synthase and glucan (1,4-alpha-), branching enzyme 1 [103,104]. HIF-dependent induction of these targets has been demonstrated to produce striking increases in glycogen deposition during hypoxic culture.

In terms of energy balance, the stimulation of energy storage by hypoxia appears paradoxical. It has been suggested to be an adaptive response to the future threat of energy starvation and the response does indeed enhance survival during adverse growth conditions [103,104]. Interestingly, however, the glycogen degradation enzyme glycogen phosphorylase (PYGL) is also induced by hypoxia, but over a longer time-scale than the synthetic enzymes [105]. Inhibition of PYGL activity was demonstrated to have striking effects on tumor cell viability, inducing senescence, and markedly impairing experimental tumor growth. Metabolic flux analysis revealed that PGYL knock-down impaired flux into the pentose phosphate pathway and generation of NADPH. The authors suggest that a critical role in redox regulation and/or provision of biosynthetic intermediates is more likely than simple lack of energy provision to explain such major effects of PYGL knock-down on cell viability [105].

#### Lipid metabolism

Abnormal lipid synthesis is yet another biosynthetic cancer phenotype that interfaces with hypoxia and HIF. Increased lipid content is a common feature of cancer cells and in some tumors large accumulations contribute to the clear cell phenotype. As with glycogen accumulation, the reasons why such large stores accumulate are not clear, though the two phenomena appear to be linked. For instance, both lipid and glycogen accumulate in clear cell renal carcinoma cells, where defective pVHL leads to constitutive activation of HIF. Whereas the clear cell phenotype is lost in normal culture medium, growth in adipogenic medium promotes accumulation of glycogen as well as lipid [106].

Despite the high energy costs (14 ATP and 7 NADPH for each molecule of the 16-carbon fatty acid, palmitate), lipid accumulation in cancer clearly involves increased *de novo* fatty acid synthesis [107]. In this process, cytoplasmic acetyl CoA is first converted to malonyl CoA by acetyl CoA carboxylase. Fatty acids are then produced by successive additions of malonyl CoA to the carbon chain, catalyzed by fatty acid synthase (FAS). Up-regulation of FAS correlates strongly with aggressive malignancy, and inhibition of FAS rapidly inhibits cancer cell proliferation, inducing cell-cycle arrest and apoptosis [108,109] (for review see [110]). Lipid synthesis potentially provides a resource for the production of new membrane and lipid signaling molecules that are important for cell proliferation. In keeping with this, synthesis of a very wide range of lipid molecules, including different types of phospholipid, cholesterol and lipid hormones, prostaglandins, leukotrienes and sphingolipids as well as fatty acids have been reported to be up-regulated in cancer. Remarkably, hypoxia appears to promote many of these synthetic pathways as well as promoting cellular lipid uptake, with interactions between lipid and hypoxia signaling pathways occurring at multiple levels [111,112]. Multiple effects on lipid synthesis gene expression patterns are apparent from large-scale pathway analyses. For instance, an extensive adipose gene expression signature is present in pVHL-defective cancer, where HIF is constitutively activated [106]. Key individual genes in lipid metabolism have also been reported to be strongly induced by hypoxia. For instance, both the cytosolic form of acetyl-CoA synthetase, which generates acetyl-CoA for fatty acid synthesis in the cytosol and FAS itself are induced in hypoxic tissue culture, while in tumors, spatial patterns of FAS expression coincide with hypoxia markers [113]. Other studies have identified hypoxia inducible genes involved in highly diverse processes in lipid biology. Examples include molecules with functions in lipid droplet formation (hypoxia-inducible protein 2), prostaglandin biosynthesis (cyclooxygenase 2), lipid signaling systems (lipoxygenase 12-lox, sphingosine kinase, SphK1) and synthetic processes (stearoyl-CoA desaturase-1, a rate-limiting enzyme in the biosynthesis of monounsaturated fatty acids) [114-117].

Though some of these genes are direct HIF transcriptional targets, in others up-regulation is mediated indirectly by an action of HIF on transcription factors that regulate lipid metabolism or adipose differentiation programs. Thus, in different settings, HIF-1 has been reported to down-regulate peroxisome proliferator-activated receptor (PPAR)-α and either to up-regulate or down-regulate PPAR-γ [118-120]. Hypoxia also interacts with many other transcriptional networks, including sterol response element binding proteins (SREBPs), DEC1/2 and GATA2/3 that act on key targets in lipid metabolism. For instance, hypoxic induction of FAS appears to involve HIF-dependent up-regulation of SREBP1 and an action of SREBP1 on the promoter of *FAS*[113].

**Figure 3:**
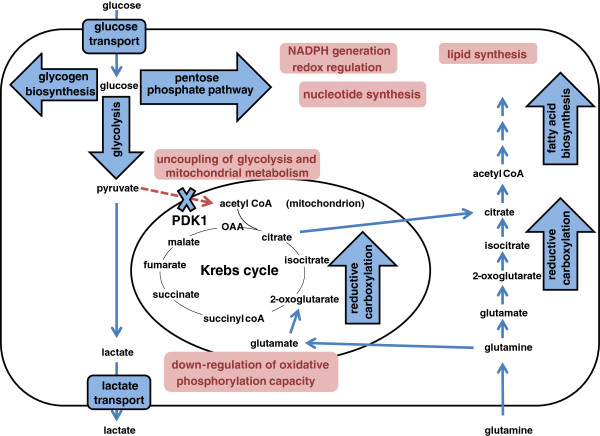
Schematic illustrating the action of HIF on multiple aspects of cellular metabolism.

Altered lipid fluxes may also occur from the effects of hypoxia on pathways that provide intermediates for lipid synthesis (Figure 3). Thus, at least in some hypoxic settings, reduced production of acetyl-CoA by pyruvate dehydrogenase (see below) impairs fatty acid synthesis from glucose. Normally, acetyl CoA that is generated in the mitochondrion by pyruvate dehydrogenase is converted to citrate by the Krebs cycle enzyme citrate synthase. When in excess, citrate can then be transported out to the cytoplasm and converted back to acetyl CoA by ATP-citrate lyase. Cytoplasmic acetyl CoA is then used in lipid biosynthesis. However, in hypoxic cancer cells, where the flow of glucose metabolites through this pathway is reduced, metabolism of glutamine can compensate to maintain the supply of cytoplasmic acetyl CoA. Metabolic flux studies have revealed that this occurs through the operation of the mitochondrial enzyme isocitrate dehydrogenase 2 (IDH2) in the reverse direction from the Krebs cycle generating citrate by reductive carboxylation of 2-OG derived from glutamine [121]. An additional cytoplasmic route of reductive carboxylation is mediated by IDH1 [122,123]. These pathways provide cytoplasmic acetyl CoA following the action of ATP-citrate lyase and metabolic flux analyses have demonstrated that the glutamine-driven reductive carboxylation is increased by hypoxia and is dependent on HIF [122]. Exactly how HIF stimulates reductive carboxylation remains unclear, but studies of HIF-α knock-down in pVHL-defective renal carcinoma cells demonstrate dependence on HIF-1α and/or HIF-2α, [121,122] leading to a reduction in intracellular citrate levels [124].

#### Mitochondrial metabolism

In a process that reflects its role in oxygen homeostasis, HIF down-regulates mitochondrial oxidative phosphorylation through a range of actions on mitochondrial metabolism and biogenesis.

In the presence of oxygen, pyruvate produced from glycolysis is converted to acetyl CoA in the mitochondrial matrix by pyruvate dehydrogenase complex (PDH), providing substrate for the Krebs cycle. Activity of the PDH is regulated by phosphorylation catalyzed by pyruvate dehydrogenase kinase (PDK) isoforms 1 to 4 and pyruvate dehydrogenase phosphatase (PDP) isoforms 1 and 2, which deactivate and activate it, respectively. HIF-1 dependent induction of PDK1 leads to PDH inhibition, thus disconnecting the Krebs cycle from glycolysis (Figure 3) [125,126].

Mitochondrial function can also be attenuated by the HIF-dependent down-regulation of the activity of several components of the electron transport chain. For example, complex 1 activity has been reported to be inhibited as a result of HIF-1-dependent activation of NADH dehydrogenase [ubiquinone] 1 alpha sub-complex 4-like 2, NDUFA4L2 [127]. Complex 2, the succinate dehydrogenase (SDH) complex (SDHA, B, C and D) also exhibits reduced activity in response to hypoxia. The exact mechanism is unclear, but is reported to involve a HIF-1 dependent reduction in SDHB protein levels through a post-transcriptional mechanism [128]. The last enzyme in the electron transport chain, cytochrome c oxidase (COX), is also a target for HIF-dependent regulation. Two of its regulatory subunits exhibit differential regulation by HIF, allowing a switch to a subunit composition that is better adapted to hypoxia: COX4-2 is a HIF transcriptional target and is up-regulated in hypoxia; whereas the COX4-1 subunit is down-regulated by an indirect mechanism achieved (at least in part) by the HIF-dependent activation of the mitochondrial LON protease that degrades COX4-1 [129]. HIF-dependent effects on mitochondrial function and the electron transport chain may also be achieved by indirect mechanisms. For instance, transcriptional activation of the micro-RNA miR-210 by HIF leads to a down-regulation of multiple targets important for mitochondrial function, including NDUFA4, SDHD, the iron-sulfur cluster assembly proteins ISCU_1/2_ and the COX assembly protein COX10 [130,131].

Finally, HIF can influence mitochondrial function through effects at the whole organelle level. Studies in the pVHL-defective renal carcinoma cell line RCC4 have shown that HIF can suppress mitochondrial respiration and mitochondrial mass through negative regulation of c-MYC, the activity of which promotes mitochondrial biogenesis [132]. Cross-talk between HIF and MYC has been defined at a number of levels, including co-operation and competition at DNA-binding sites, non-DNA binding interaction between specific HIF-α isoforms and MYC subunits, and interactions mediated by expression of the HIF target gene *MXI-1*, which represses c-MYC activity [132]. In addition, the HIF target gene *BNIP3* contributes to reduced mitochondrial number through increased mitochondrial autophagy [133].

Overall, these effects of HIF cause an adaptive shift away from mitochondrial respiration. Together with preservation of biosynthetic capacity, through the generation of citrate by reductive carboxylation, this conveys survival advantages in hypoxia. HIF-1α defective cells are unable to down-regulate oxygen consumption in the face of hypoxia and show impaired survival in hypoxia, at least in part due to an enhanced production of reactive oxygen species [129].

## Conclusions

Overall these studies have defined multiple means by which HIF is up-regulated in cancer (Figure 2) and multiple effects of HIF activation on metabolism in cancer (Figure 3). Furthermore, although HIF acts (directly or indirectly) on genes encoding a wide range of enzymes that contribute to metabolic dysregulation in cancer, many of these enzymes are also direct targets of the oncogenic pathways. For instance, activation of PI3K/PTEN/AKT signaling activates HIF and, hence, indirectly activates transcription of genes encoding multiple glycolytic enzymes. However, AKT activation also directly up-regulates glucose metabolism via a range of other mechanisms, including the increased expression [134] and trafficking of glucose transporters to the cell membrane [135,136], increased expression of glycolytic enzymes and altered phosphorylation of regulatory components, such as PFKFB2 [137-139]. The parallel operation of these complex pathways makes the non-redundant role of any one molecular connection difficult to predict. Unsurprisingly, given that HIF is primarily a transcription factor, the large majority of studies have focused on the effects on gene expression and there are relatively few studies of the effect of HIF on specific metabolic fluxes. However, key enzyme activities are regulated at many levels other than gene expression, and altered metabolism requires coordinated changes in the function of many enzymes. Therefore, overall effects on metabolic fluxes are difficult to derive from the measurement of gene expression levels, and more direct assessments of the effects of HIF activation on fluxes through different metabolic pathways would be useful. Despite this caveat, it is clear that HIF contributes to very many aspects of dysregulated metabolism in cancer cells. Given the diversity of these effects, an important, but as yet incompletely answered question, concerns the extent to which the effects of HIF activation and oncogenic activation coincide on the same metabolic pathways.

The most obvious difference between the effects of HIF activation and those of oncogenic pathways is apparent in the actions on mitochondrial function. HIF activation down-regulates mitochondrial biogenesis and mitochondrial metabolism, whereas oncogenic pathways generally have the reverse effect, promoting mitochondrial metabolic capacity by multiple mechanisms (reviewed in [140,141]). As mitochondrial metabolism is the main component of cellular oxygen consumption, this action of HIF to reduce oxygen consumption in the face of hypoxia can be rationalized in terms of the central role of HIF in maintaining oxygen homeostasis and it has been proposed that, in this setting, HIF acts to tune the metabolic dysregulation in cancer in accordance with availability of oxygen.

In contrast with effects on mitochondrial metabolism, a wide range of other metabolic functions manifest close similarities between the effects of HIF activation and those of activated oncogenic pathways. The concordance between the isoforms of glycolytic enzymes that are up-regulated by HIF, and those that are targeted directly by oncogenic pathways is striking. Also remarkable is the concordance between the activation of biosynthetic pathways involving excess glycogen and lipid production, by both HIF and activated oncogenic pathways. Enhanced production of ATP by glycolysis in hypoxic cells is clearly consistent with the maintenance of oxygen homeostasis. However, the pattern of glycolytic enzyme isoform activation that is induced both by HIF and by oncogenic pathways is not such as to maximize ATP production, and a large body of evidence suggests that its prime function is the supply of biosynthetic intermediates. Similarly, activation of both glycogen synthesis and lipid biosynthesis (again by both HIF and oncogenic pathways) is hypothesized to reflect the needs of proliferating cells for biosynthetic intermediates (reviewed in [110,142]). Since these processes neither reduce oxygen demand, nor increase oxygen supply, it is not at all clear how these effects relate to the concept of HIF as a regulator of oxygen homeostasis. Thus it may be necessary to revise thinking on the prime function of the HIF system to include general support of growth/repair pathways (perhaps as a response to hypoxic tissue damage) as opposed to more restricted growth pathways, such as angiogenesis or erythropoiesis, that directly enhance oxygen delivery.

Whatever the explanation, a large body of evidence implicates HIF in the up-regulation of biosynthetic pathways that support the growth of cancer cells while multiple genetic and micro-environmental alterations promote the up-regulation of HIF in cancer. Given the compelling associations between HIF activation, aggressive phenotypes and adverse prognosis across different types of cancer, it is tempting to conclude that activation of HIF is not only a major contributor to the cancer phenotype, but that it is causally implicated in cancer progression. Surprisingly, while mechanistic analysis strongly supports the first of these statements, there is a striking absence of human genetic evidence to support the second. As outlined above, cancer genome analyses have not yet defined clusters of mutations typical of oncogenic activation in genes encoding any HIF subunit; to date the clearest evidence for mutational selection on HIF in cancer is an excess of inactivating mutations in HIF-1α in the context of pVHL-defective renal cancer (reviewed in [143]).

### The 'co-selection’ hypothesis

We have previously drawn attention to this paradox and argued that the extraordinarily strong association between HIF activation and aggressive cancer most likely reflects the existence of 'hard-wired’ pathways that operate physiologically to link tissue growth to oxygen supply and are, therefore, 'co-selected’ by oncogenic pathways that drive cellular proliferation directly [144]. An important implication of this 'co-selection’ hypothesis is that the very extensive connections of the HIF transcriptional cascade will all be 'co-selected’ into the cancer phenotype (by both oncogenic or micro-environmental activation of HIF), irrespective of whether they are promoting or restricting cancer growth. Although many aspects of HIF activation may contribute to cancer development, the existence of cytostatic responses that form part of the physiological response to hypoxia may have the reverse effect, possibly accounting for the observation that HIF-1α displays some of the characteristics of a tumor suppressor, at least in the context of pVHL-defective renal cancer [87]. Thus 'co-selection’ of massive 'hard-wired’ HIF pathways into the cancer phenotype may incur a substantial fitness penalty and the existence of tight prognostic associations with phenotype does not necessarily imply causality. In considering the interface between HIF activation and metabolic dysregulation in cancer, an important question is how far do similar arguments extend to the massively complex HIF-dependent and HIF-independent pathways that underpin metabolic dysregulation in cancer?

A key advance in the understanding of cancer metabolism has been the recognition that dysregulated metabolic pathways are not simply responding to error signals, such as metabolite depletion created within the tumor mass, but (as with HIF), are directly connected to oncogenic signaling pathways [145,146]. Furthermore, like HIF, a similar genetic paradox exists. Although gene amplification events have been described for a number of metabolic enzymes that are over-expressed or activated in cancer [146], for most metabolic pathways whose up-regulation is strikingly associated with aggressive cancer, there is an apparent paucity of mutations that directly activate the pathway. Interestingly, this paradox has been brought into even sharper focus by the striking mutational profiles that do exist in very specific 'metabolic’ tumor suppressor genes (that is, *FH* and *SDH*) that are inactivated in specific rare cancer syndromes [30-32], and in the IDH1 and 2 oncogenes.

**Figure 4:**
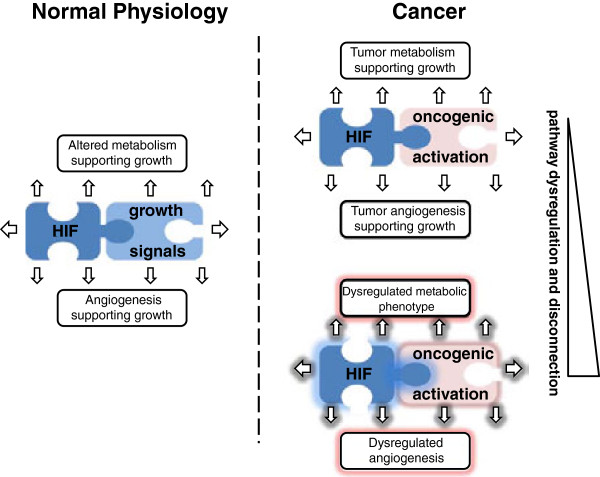
**Schematic illustrating 'hard-wired’ interconnections among growth promoting signals, HIF and metabolic/angiogenic pathways.** During physiological growth these pathways are tightly regulated (left panel). In oncogenesis, increased growth activates the 'hard-wired’ connections to generate the metabolic and angiogenic characteristics of cancer (upper right panel). Because different oncogenic signals have quantitatively and qualitatively different connections with hypoxia inducible factor (HIF) and metabolic/angiogenic pathways, dysregulated stochastic activation of oncogenic signaling by somatic mutation in cancer has the potential to 'co-select’ grossly disorganized metabolic and angiogenic phenotypes (lower right panel).

The mutational profile of IDH1 and 2 is particularly informative. Multiple different substitutions are observed at highly specific substrate binding residues in each gene, but not elsewhere. These mutations are heterozygous and conform to the classical expectations of an oncogene (acting dominantly to promote cancer) and strong evidence has been assembled for their oncogenic action through the production of 2-HG [40]. Evidence for the proposed action of 2-HG on 2-OG-dependent dioxygenases that are involved in epigenetic regulation or other potential oncogenic pathways has been reviewed elsewhere [147]. What is important for the current discussion is that the IDH1/2 mutational profile acts as a striking positive control, emphasizing the extraordinary capacity of human cancer to 'purify’ mutations that do provide selective advantage. Against this background, the relative lack of activating mutations in enzymes belonging to other metabolic pathways that are up-regulated in cancer is striking. As with HIF, this contrasts markedly with the numerous connections that have been defined between metabolic enzymes and mutated oncogenes. Thus, it appears likely that co-selection of 'hard-wired’ metabolic pathways that exist physiologically to support dividing cells may therefore extend to both HIF-dependent and HIF-independent pathways underpinning dysregulated metabolism in cancer (Figure 4). Though multiple intervention studies support the positive contributions of metabolic dysregulation to cancer growth and, hence, the possibility that these pathways represent potential targets for anti-cancer treatment [105,148], the 'co-selection’ hypothesis implies that this cannot be inferred directly from the strong association with aggressive malignancy, and that direct testing in the context of specific cancers will be required.

In an extension of this argument, co-selection may explain one otherwise puzzling aspect of dysregulated cancer metabolism, the occasional generation of bizarre cellular phenotypes by apparently excessive accumulation of energy stores. As outlined above, increased glycogen and lipid biosynthesis are common features of cancer cells. While activation of these processes can be rationalized in terms of provision of biosynthetic intermediates necessary for cellular growth, it is much more difficult to understand how the massive accumulation of glycogen and lipid associated with the 'clear cell’ phenotype in certain cancers assists cancer growth. Similar arguments apply to cancer-associated angiogenesis. The entrainment of an oxygen supply through effective angiogenesis is clearly important for cancer growth, but the bizarre and ineffective excessive angiogenic activity that is often observed in tumors is more difficult to understand, particularly under the hypothesis that up-regulation of angiogenesis is directly selected. We propose that both may represent the effects of 'co-selecting’ circuits that are 'hard-wired’ to oncogene activation to support the needs of growing tissues. Under physiological conditions these pathways are tightly regulated so that enhanced 'metabolic’ and 'angiogenic’ activity is well coordinated with cellular growth. In cancer these processes are also, in general, fairly well coordinated, so as to entrain broadly functional metabolic and angiogenic support pathways. However, review of the mechanisms and pathways leading to HIF activation in cancer reveals mechanisms that are qualitatively and quantitatively diverse. Thus, the stochastic activation of individual oncogenic pathways by genetic mutations conferring selection through cell autonomous advantage also has the potential to create disorganized and chaotic activation of metabolic and angiogenic pathways, dependent on the nature of the pre-existing link between the affected oncogene and metabolic/angiogenic pathways. For instance, whereas under some circumstances pathway activation may be inadequate to support cancer growth (for example, poorly angiogenic tumors), under other circumstances (for example, massive stores of glycogen and lipid and exaggerated angiogenesis associated with powerful and direct activation of HIF in pVHL-defective tumors), it is excessive (Figure 4).

In summary, work on the HIF pathway has revealed massively complex interactive connections between cancer growth and HIF activation and between HIF activation and cancer phenotypes. We argue that one of the most important and, so far, under-appreciated implications of these findings is the need to consider the implication of co-selecting massive hard-wired physiological pathways as cancer progresses. Though we have reviewed this consideration from the perspective of HIF (where the ease with which cells can be exposed to the physiological stimulus of hypoxia has led to a rapid appreciation of the enormous complexity of the pathway), similar considerations are likely to apply to other pathways that may be activated by micro-environmental stress or genetic mutation in cancer.

## Abbreviations

2-HG: 2-hydroxyglutarate; 2-OG: 2-oxoglutarate; AMPK: AMP-activated protein kinase; bHLH-PAS: Basic-Helix-Loop-Helix Per-AHR/ARNT/Sim; CAD: C-terminal activation domain; COX: Cytochrome C oxidase; eEF2: Eukaryotic translation elongation factor 2; EGLN: Egg-laying defective like; eIF2α: Eukaryotic translation initiation factor 2 alpha; FAS: Fatty acid synthase; FDG-PET: Fluorodeoxyglucose in positron emission tomography; FH: Fumarate hydratase; FIH: Factor inhibiting HIF; GSK-3b: Glycogen synthase kinase-3 beta; IDH: Isocitrate dehydrogenase; HDAC: Histone deacetylase; HIF: Hypoxia inducible factor; MDM2: Mouse double minute 2 homolog; mTORC: Mammalian target of rapamycin complex; NAD: Nicotinamide adenine dinucleotide; NADPH: Nicotinamide adenine dinucleotide phosphate; NF-kB: Nuclear factor kappa B; OAA: Oxaloacetate; PCAF: p300/CBP associated factor; PDH: Pyruvate dehydrogenase complex; PDK: Pyruvate dehydrogenase kinase; PDP: Pyruvate dehydrogenase phosphatase; PERK: Protein kinase-like Endoplasmic Reticulum Kinase; PFK-1: 6-phosphofructo-1-kinase; PHD: Prolyl hydroxylase domain; PI3K: Phosphatidylinositide 3-kinase; PKM1/2: Pyruvate kinase isoform M1/2; PPAR: Peroxisome proliferator-activated receptor; PTEN: Phosphatase and tensin homolog; PYGL: Glycogen phosphorylase; VHL: Von Hippel-Lindau; REDD1: Regulated in development and DNA damage responses 1; SDH: Succinate dehydrogenase; shRNA: Short hairpin RNA; SIRT: Sirtuin; SREBP: Sterol response element binding protein; TSC: Tuberous sclerosis; VEGF: Vascular endothelial growth factor

## Competing interests

The authors declare no financial competing interests.

## Authors’ contributions

PJR conceived the review, revising and critically assessing the content. NM was responsible for critically reviewing the literature and drafting sections and figures. Both authors read and approved the final manuscript.
